# Performance Evaluation of Smartphone Inertial Sensors Measurement for Range of Motion

**DOI:** 10.3390/s150923168

**Published:** 2015-09-15

**Authors:** Quentin Mourcou, Anthony Fleury, Céline Franco, Frédéric Klopcic, Nicolas Vuillerme

**Affiliations:** 1 University of Grenoble-Alpes, AGIM, La Tronche 38700, France; E-Mails: Celine.Franco@agim.eu (C.F.); Nicolas.Vuillerme@agim.eu (N.V.); 2 University of Lille, F-59000 Lille and Mines Douai, URIA, Douai F-59508, France; E-Mails: Anthony.Fleury@mines-douai.fr (A.F.); Frederic.Klopcic@mines-douai.fr (F.K.); 3 Institut Universitaire de France, Paris 75000, France; 4 LAI Jean-Raoul Scherrer, University of Geneva, 1206 Geneva Switzerland/University of Grenoble Alpes, Saint-Martin-d'Hères 38041, France; 5 Laboratory for Ergonomics and Work-related Disorders, Center for Sensory-Motor Interaction (SMI), Department of Health Science and Technology, University of Aalborg, Aalborg 9220, Denmark

**Keywords:** smartphone sensing, IMU, Kalman filter, validation

## Abstract

Over the years, smartphones have become tools for scientific and clinical research. They can, for instance, be used to assess range of motion and joint angle measurement. In this paper, our aim was to determine if smartphones are reliable and accurate enough for clinical motion research. This work proposes an evaluation of different smartphone sensors performance and different manufacturer algorithm performances with the comparison to the gold standard, an industrial robotic arm with an actual standard use inertial motion unit in clinical measurement, an Xsens product. Both dynamic and static protocols were used to perform these comparisons. Root Mean Square (RMS) mean values results for static protocol are under 0.3° for the different smartphones. RMS mean values results for dynamic protocol are more prone to bias induced by Euler angle representation. Statistical results prove that there are no filter effect on results for both protocols and no hardware effect. Smartphones performance can be compared to the Xsens gold standard for clinical research.

## 1. Introduction

Smartphones have become an unavoidable tool in developed countries and even an important part of life. There were more than a billion smartphones sold worldwide in 2014, a 23% increase in shipments between full year 2013 and 2014 [1]. The sharp decline in the price of mobile equipment allows growth in emerging markets. Although they are becoming more and more affordable, mobile phones remain powerful tools composed of a processor, a graphics chip, advanced connectivity and an inertial motion unit (IMU), with a 3D-accelerometer, magnetometer and gyroscope as standard features. Moreover, smartphones contain more technology such as a screen display, an audio system or a haptic feedback system that enables interaction with the user of the device. To combine and use all these functionalities, smartphones are able to run specific software, called “applications”.

With all these features, smartphones have widely been used as tools for scientific and clinical research, especially in the healthcare and physical activity monitoring fields. For example, smartphone could be used to assess range of motion and joint angle measurement for postural and gait control [2] or for joint goniometry [3]. These applications are designed to provide accurate and reliable range of motion measurements compared to the standard tools. Current smartphone applications mainly use algorithms provided by manufacturers, which process a combination of three sensors, the accelerometer, the gyroscope and the magnetometer to compute an angle value. Although they have been directly compared to standard tools, angle measurement from a smartphone in the context of static and dynamic measurements has not been investigated yet. To the best of our knowledge, the scientific literature does not provide assessments of the performance of the tool and its sensors for the particular context of clinical measurement against a very specific gold standard, such as Kuka robot. In addition, evaluation of the performance of algorithms for calculating angles incorporated into these phones has also not yet been specifically performed. In this context, the goal of this paper is to propose an evaluation of different smartphone sensors performance and different manufacturer’s algorithm performance with the comparison to a gold standard, an industrial robotic arm and with a standardly used IMU in clinical measurement, an Xsens product. Our hypothesis is that the smartphone is able to perform measurements that are accurate enough to be used in clinical settings, in replacement of specific devices. These comparisons will justify or forgive the use of Smartphone sensors and software for clinical measurement. Furthermore, with the comparison of static and dynamic conditions, this study is intended to cover all types of clinical movements that could be performed during assessment and rehabilitation.

The remaining of this paper is organized as follows. Section 2 describes related works on performance evaluation of smartphone inertial sensors. Section 3 describes the materials and methods, with a description of our evaluation approach in terms of protocol and statistical analysis. Then, the effect of position and filter, and performance comparison are presented in Section 4 and discussed in Section 5. Conclusions are finally drawn in Section 6.

## 2. Related Works 

All sensor-based applications bring innovation in research and will lead to cognitive-phone, which will infer human behavior and context to give specific help to the patient [4]. This evolution could be characterized as Ambient Intelligence, the basic idea of which is to enrich the environment with smart technologies [5]. Ambient Intelligence systems have to be sensitive, responsive, adaptive, transparent, ubiquitous and intelligent. Among these smart technologies, the smartphone could take a significant place and play a major role insofar as it is becoming increasingly used in everyday life. Nowadays, sensor fusion algorithms integrated in smartphones can be used for clinical research, to sense human body motions in Activities of Daily Living (ADL) [6] to assess range of motion and joint angle measurement for postural and gait control [2], for joint goniometry [3], and even for fall detection [7]. The use of a smartphone for fall detection is an example of the multiple possible uses of these different sensors. An accelerometer is used by all smartphone fall detection and prevention solutions but their dynamic ranges are often insufficient [8]. Thus, the quality of sensors is crucial when using a smartphone for clinical purposes. To perform angle measurements, such as range of motion, an accelerometer can be used alone to measure tilt angle, but it is only reliable when the smartphone is static. To get accurate angle measurement in dynamic, Williamson and Andrews combined accelerometers with gyroscopes, which are insensitive to the influence of gravity [9]. Repeated sit-to-stand and stand-to-sit movements were performed to lead to that conclusion. Then, to measure the horizontal component of orientation, Kemp *et al.* combined accelerometers and magnetometer to monitor body position [10,11,12]. Moreover, we find that, in the literature concerning the angular measurement using Smartphone, proofs of concept only verify the concordance of the measurements in specific contexts and not the global performance and accuracy of smartphones sensors (checking for instance that they can detect sit-to-stand or that the measurement of the movement gives relatively the same information in terms for instance of Root Mean Square (RMS) through different trials). To the best of our knowledge, the existing literature does not provide any information regarding the performance of the inertial sensors and the reliability of the algorithms provided by the smartphone manufacturer to perform angle measurements that are clinically acceptable. The present study was hence designed to address this lack of information. Our goal was to propose an assessment of different smartphone sensors and different manufacturer’s algorithm performances. It evaluates three best-selling smartphones (Apple iPhone 4, Apple iPhone 5S and Samsung Galaxy Nexus), which are still widely used in research, teaching, and in industrial applications. Their sensors and embedded software are also available in a wide variety of other smartphones on the market. To perform angle measurement, we used some algorithms that are state-of-the art and are available for all scientific community [13] but also the algorithms that are given by the two major manufacturers of smartphone inside the Software Development Kit (SDK) of their Operating System. Such evaluations are crucially needed to allow or not the use of smartphones internal sensors and manufacturer’s or Attitude and Heading Reference System (AHRS) algorithms implemented on smartphones to perform clinical angle measurement compared to specific tool, which are more precise than standard clinical tools.

## 3. Materials and Method

### 3.1. Material

#### 3.1.1. Smartphones

The smartphone market can be complex because it includes different manufacturers and different software providers. In order to cover the majority (in terms of number of units sold) of this market, we have chosen to select three different and representative smartphones from the two largest sellers (Apple and Samsung) and which contains different sensors and software.

The first Smartphone tested is an Apple iPhone 4 equipped with (1) a 3D accelerometer (ST-Microelectronics, LIS331DLH, Geneva, Switzerland), (2) an integrated 3D gyroscope (ST-Microelectronics, L3G200D, Geneva, Switzerland), and (3) a 3D magnetometer (Asahi Kasei Microdevices, AKM8975, Nobeoka, Japan). This device runs, for these tests, iOS 7 operating system with our homemade software. This software is nothing more than a simple graphical interface that collects the data using different algorithms (embedded or given by the OS) using selected frequency and conditions. All data are saved in a Comma Separated Values (CSV)-like file for future use and statistics. 

The second Smartphone tested is an Apple iPhone 5S equipped with (1) a 3D accelerometer (Bosch Sensortec BMA220, Gerlingen, Germany), (2) an integrated 3D gyroscope (ST-Microelectronics, L3G4200DH, Geneva, Switzerland), and (3) a 3D magnetometer (AKM, AK8963, Tokyo, Japan). The device also runs iOS 7 operating system and our homemade software.

The third Smartphone tested is a Samsung Galaxy Nexus equipped with (1) a 3D accelerometer (Bosch Sensortec BMA220, Gerlingen, Germany), (2) an integrated 3D gyroscope (InvenSens, MPU-3050, San Jose, CA, USA), and (3) a 3D magnetometer (Yamaha, YAS530, Shizuoka, Japan). The device runs Android 4.3 Jelly Bean operating system with our homemade software.

#### 3.1.2. Xsens

Xsens IMU are commonly used in motion sensing applications, and as the gold standard for much scientific research [14,15,16,17]. Motion Trackers MTx are the selected devices for this experiment. It contains all solid-state miniature MEMS inertial sensors inside (accelerometer, magnetometer and gyroscope). Their static accuracy for roll and pitch is under 0.5° and for yaw under 1° according to the manufacturer. Data are collected using the given Xsens MT Software.

#### 3.1.3. Robot

**Figure 1:**
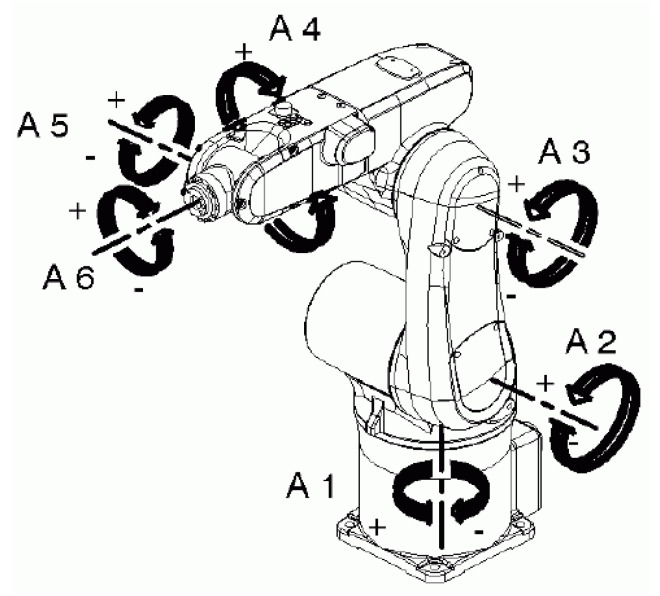
KR5-SIXX-R650 Axis. Provided by KR 5 sixx R650, R850 Specification manual.

The robot used for the experiment is a KR5-SIXX-R650 manufactured by Kuka (Figure 1). This robot is a 6-axis jointed-arm robot made of cast light alloy. It consists, from bottom to top, of a base frame with a rotated column, then a link arm, the arm and finally an in-line wrist. We used, for pitch, the A5 axis, for roll the A4 axis and for yaw the A6 axis. The range of motion is about ±120° for A5, ±190° for A4 and ±358° for A6.The speed with rated payload of 5 Kg is about 410°/s for A4 and A5 and 660°/s for the last axis. Repeatability accuracy is ±0.02 mm according to the manufacturer [18].

### 3.2. Angle Estimation

**Figure 2:**
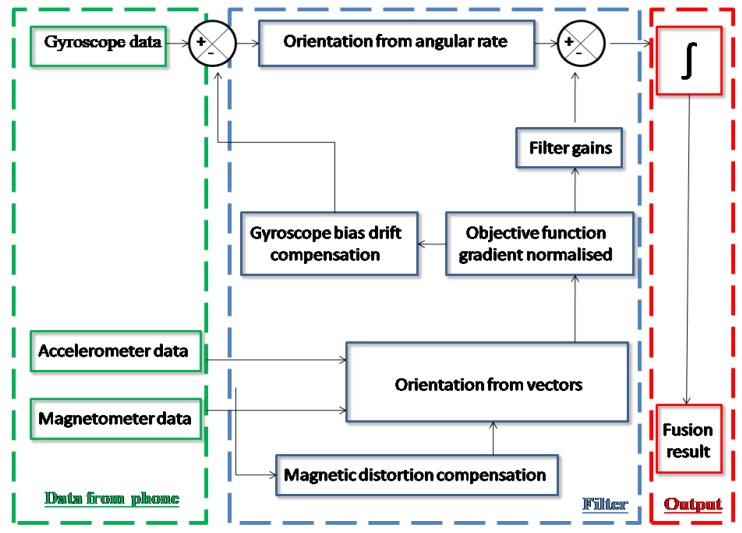
Block diagram representation of a common orientation filter using accelerometer, magnetometer and gyroscope data.

From accelerometer, magnetometer and gyroscope, we can obtain raw measurements for acceleration, ambient geomagnetic field and angular velocity, respectively. However, to compute orientation estimation, none of these sensors bring noiseless information. With these three sensors, we are able to build an AHRS (Attitude and Heading Reference System) in which each sensor will compensate for the bias induced by others. Thus, the Earth gravitational and magnetic fields, respectively, measured by accelerometer and magnetometer, will be merged with angular velocity from gyroscope to compute a single and complete estimate of orientation angles. This solution is provided by orientation filter, among which we can mention the Complementary filter [19], the Kalman filter [20], the Mahony filter [21] and the Madgwick filter [13]. Orientation estimation is similar to evaluation of the kinematic equation for the rotation of the device. Filter task is to compute, from a given rotation, an improved estimated attitude. Thus, filter computes estimated attitude as the rate of change of original attitude measured by gyroscope with the magnitude of the gyroscope measurement error, which is removed in the direction of the estimated error computed from accelerometer and magnetometer measurements [13]. To improve it, magnetic distortion and gyroscope bias drift has to be compensated. The approaches of Kalman, Madgwick and Mahony differ on the resolution of these biases. For example, Mahony uses a proportional and integral controller to correct gyroscope bias, while Madgwick uses only a proportional controller. Block diagram representation for common orientation filter is presented in Figure 2. All these three filters use a quaternion representation. It is a four-dimensional complex number representing the orientation of the device. Although easier to calculate and more efficient, quaternion are less human understandable than Euler angles, which is the representation used in kinematic and clinical field. Compared to quaternion, Euler angles are subject to ambiguity and gimbal lock, two known problems of this representation that have been taken into account in out protocol of measurement. Gimbal lock is a singularity that appears when two axes of the object have parallel orientation. It causes the loss of one degree of freedom and thus a measurement inaccuracy. In order to be as comprehensive as possible, we have chosen, for each device, to compare the filter proposed by the manufacturer with both Madgwick and Mahony filters.

### 3.3. Filters Implementation Algorithms

In order to collect data from the different smartphones, we built, for each operating system, a dedicated application to collect, at a 50 Hz frequency, acceleration force in m/s², geomagnetic field in µT and angular rate in rad/s for the three physical axes of device (*x*, *y*, *z*). In addition, we collected, at the same frequency, Euler angles values from filters provided by manufacturers, using the methodology described in the respective documentations. Other filters (Madgwick and Mahony) are then calculated from the raw values and converted from quaternion to Euler angles following algorithms described in the next section. Thereby, our dedicated application is able to collect orientation data from three different filters: manufacturer filter, Madgwick algorithm and Mahony filter. All filters use the same raw data provided by the smartphone’s internal sensors, and the raw and filtered values are saved at the same time. Computation of Madgwick and Mahony filters with Xsens raw data were performed with Matlab software (Mathworks, MA, USA) using the source code provided by Madgwick [13].

#### 3.3.1. Mahony Filter

To implement Mahony Filter on iOS or Android operating systems, sample code provided by Madgwick in C and MatLab languages were used and converted. In both systems, we created a method that takes as arguments all raw data from gyroscope, accelerometer and magnetometer and the previous estimate of orientation. This method is executed at the sampling rate of 50 Hz using the dedicated methods to obtain precision timers for each OS. The following formulas used the same notation as Madgwick [13]. $\begin{array}{c} A\\ B \end{array}\hat{q}$ describes the orientation of frame B relative to frame A and $\begin{array}{c} A \end{array}\hat{v}$ is a vector described in frame A. Algorithm begins with the normalization of accelerometer measurement and magnetometer measurement. To perform normalization and keep efficiency of computation, the fast inverse square root is used to product our normalized unit vector for both measurements as Equation (1):

(1) $$\hat{v}\;=\;1/\sqrt{v_{x}^{\text{2}}+v_{y}^{2}+v_{z}^{2}}$$

where $v_{x}$, $v_{y}$ and $v_{z}$ are axis of frames from sensors. Then, $v_{x}$, $v_{y}$ and $v_{z}$ are multiplied by $\hat{v}$. After that, the measured direction of the earth’s magnetic field in the earth frame, $\begin{array}{c} E \end{array}{\hat{h}}_{t}$, is calculated as suggested and described [13]. It is the quaternion product of the previous estimate of orientation with the normalized magnetometer measurement and with the quaternion conjugate of the previous estimate of orientation, Equation (2). The ⊗ operator denotes a quaternion product and the hat on h, $\hat{h}$, denotes a normalized vector of unit length.

(2) Eh^t = SEq^ est,t−1 ⊗ mst ⊗ SEq^*est,t−1

The effect of an erroneous inclination of the measured direction of the Earth magnetic field is corrected using $\begin{array}{c} E \end{array}{\hat{b}}_{t}$ which is the normalization of $\begin{array}{c} E \end{array}{\hat{h}}_{t}$ to have only components in the earth frame x and z axes Equation (3).

(3) $$\begin{array}{c} E \end{array}{\hat{b}}_{t}\;=\;\left[\text{ 0}\;\sqrt{h_{x}^{\text{2}}+h_{y}^{2}}\;0\;h_{z}\right]$$

Then, the estimated direction of gravity Equation (5) and magnetic field Equation (6) are calculated as follows, using Equation (4) as quaternion definition:

(4) $$\begin{array}{c} S\\ E \end{array}\hat{q}\;=\;\left[q_{\text{1}}\;q_{\text{2}}\;q_{\text{3}}\;q_{\text{4}}\right]$$

(5) $$\begin{array}{c} E \end{array}{\hat{v}}_{est,t}\;=\;\left[\text{2}\times \left(q_{\text{2}}\times q_{\text{4}}-q_{\text{1}}\times q_{\text{3}}\right)\;2\times \left(q_{\text{1}}\times q_{\text{2}}+q_{3}\times q_{\text{4}}\right)\;q_{\text{1}}^{\text{2}}-q_{\text{2}}^{\text{2}}-q_{\text{3}}^{\text{2}}+q_{4}^{2}\right]$$

(6) $$\begin{array}{c} E \end{array}{\hat{w}}_{est,t}\;=\;\left[\begin{array}{ll} \text{2}\times b_{\text{2}}\times \left(\text{0.5}-q_{3}^{2}-q_{\text{4}}^{\text{2}}\right)+\text{2}\times b_{\text{4}}\times \left(q_{\text{2}}\times q_{\text{4}}-q_{\text{1}}\times q_{\text{3}}\right) & 2\times b_{\text{2}}\times \left(q_{\text{2}}\times q_{\text{3}}-q_{\text{1}}\times q_{\text{4}}\right)+\text{2}\times b_{\text{4}}\times \left(q_{\text{1}}\times q_{\text{2}}+q_{3}\times q_{\text{4}}\right)\\ \text{2}\times b_{\text{2}}\times \left(q_{\text{1}}\times q_{\text{3}}+q_{2}\times q_{\text{4}}\right)+\text{2}\times b_{\text{4}}\times \left(\text{0.5}-q_{\text{2}}^{\text{2}}-q_{\text{3}}^{\text{2}}\right) & \end{array}\right]$$

Next, the computed and estimated error is the sum of cross product between estimated direction and measured direction of field vectors Equation (7).

(7) $$\begin{array}{c} E \end{array}{\hat{e}}_{est,t}\;=\;a_{t}\;\times \;\begin{array}{c} E \end{array}{\hat{v}}_{est,t}\;+\;m_{t}\;\times \;\begin{array}{c} E \end{array}{\hat{w}}_{est,t}$$

After that, the specificity of this filter is to apply an integral (calculated with Equation (8)) and a proportional controller to correct gyroscope bias, Equation (9), where sp is the sample rate (50 Hz).

(8) $$i_{t}\;=\;i{\;}_{t-\text{1}}\;+\;\begin{array}{c} E \end{array}{\hat{e}}_{est,t}\;\times \;sp$$

(9) $$\begin{array}{c} E \end{array}{\hat{g}}_{t}\;=\;\begin{array}{c} E \end{array}\hat{g}{\;}_{t-\text{1}}\;+\;k_{p}\;\times \;\begin{array}{c} E \end{array}{\hat{e}}_{est,t}\;+\;k_{i}\;\times \;i_{t}$$

Finally, the rate of changes of quaternion Equations (10) and (11) is integrated, normalized and converted to Euler in order to compare these measurements with Euler angle measurements from Madgwick and manufacturer’s filters.

(10) $$qDot\;=\;0.5\;\times \;\begin{array}{c} S\\ E \end{array}\hat{q}{\;}_{est,t-\text{1}}\;⊗\;\begin{array}{c} E \end{array}{\hat{g}}_{t}$$

(11) $$\begin{array}{c} S\\ E \end{array}\hat{q}{\;}_{est,t}\;=\;\begin{array}{c} S\\ E \end{array}\hat{q}{\;}_{est,t-\text{1}}\;+\;qDot\;\times \;sp$$

#### 3.3.2. Madgwick Filter

To implement the Madgwick filter on Android and iOS operating systems, sample code provided by Madgwick in C and MatLab languages are also used. A method that takes as arguments all raw data from gyroscope, accelerometer and magnetometer and the previous estimate of orientation was created. This method is executed at the sampling rate of 50 Hz, as Mahony and manufacturer’s methods. The algorithm begins with the normalization of accelerometer and magnetometer measurements, using the same methodology as Section 3.3.1. The measured direction of the Earth magnetic field in the earth frame and the effect of an erroneous inclination of the measured direction Earth magnetic field are calculated using this same methodology, too. Then, a gradient descent algorithm corrective step is used, as described in [13]. This is the specificity of this filter compared to Mahony filter. The gradient descent algorithm corrective step yields to the simplified objective function and Jacobian defined by Equations (12) and (13).

(12) fb(q^ES,b^E,m^S)=[2bx(0.5−q32−q42)+2bz(q2q4−q1q3)−mx2bx(q2q3−q1q4)+2bz(q1q2+q3q4)−my2bx(q1q3+q2q4)+2bz(0.5−q22−q32)−mz]

(13) Jb(q^ES,b^E)=[−2bzq32bzq4−4bxq3−2bzq1−4bxq4+2bzq2−2bxq4+2bzq22bxq3+2bzq12bxq2+2bzq4−2bxq1+2bzq32bxq32bxq4−4bzq22bxq1−4bzq32bxq2]

To provide a unique orientation, Equations (14) and (15) combines the measurement of gravity and the Earth’s magnetic field.

(14) fg,b(q^ES,a^S,b^E,m^S)=[fg(q^ES,a^S)fb(q^ES,b^E,m^S)]

(15) Jg,b(q^ES,b^E)=[JgT(q^ES)JbT(q^ES,b^E)]

Next step is the multiplication of Equations (14) and (15), and a normalization of the result. Then, to apply feedback step (estimation of the error for further more efficient correction), this result is multiplied by the algorithm adjustable parameter, a proportional controller, which represent the gyroscope measurement error expressed as the magnitude of a quaternion derivative. Then, to compute rate of change, result is subtracted from the rate of change of orientation measured by the gyroscopes (Equation (16)).

(16) qDot=0.5×q^ESest,t−1⊗g^Et−β×sT

Where β is the proportional controller and $s^{T}$ is the transpose result of the multiplication of Equations (14) and (15). Finally, rate of change of quaternion is integrated, normalized and converted to Euler angles.

### 3.4. Method

#### 3.4.1. Global Methodology

**Figure 3:**
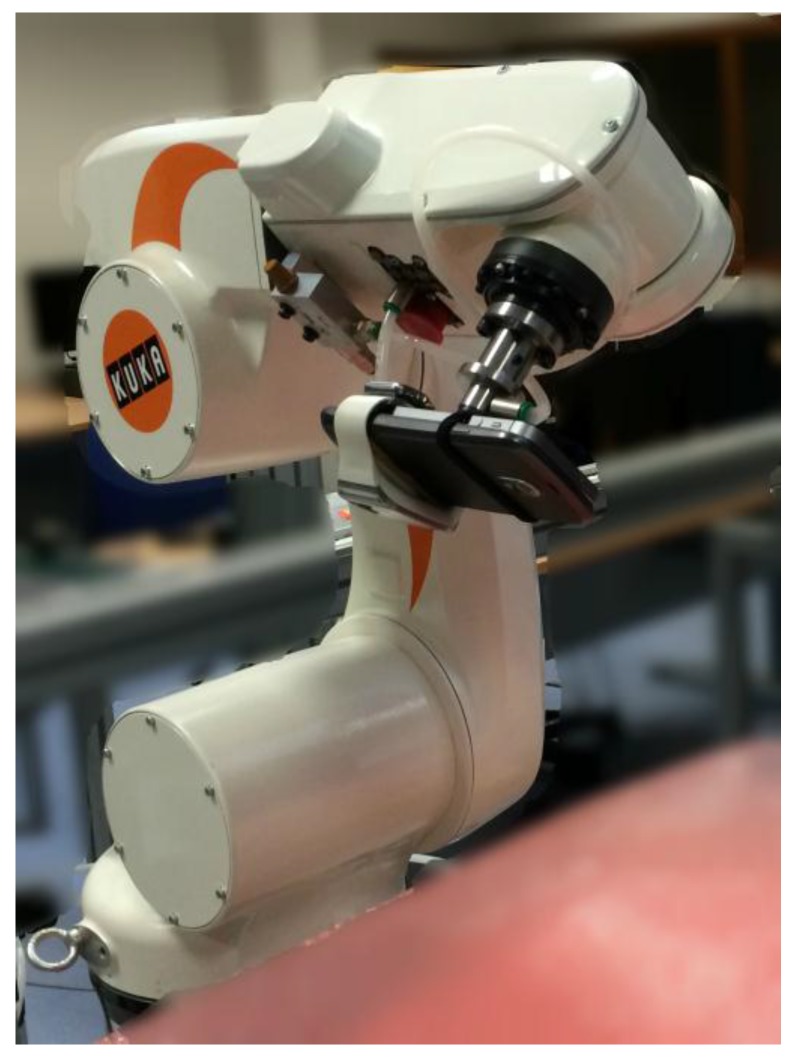
Picture showing the two smartphones superimposed mounted on the robotic arm.

To perform evaluation of smartphone sensors hardware and angle measurement algorithm precision, we reproduced some angular movements on two axes, pitch and roll. All tested devices were fixed on the Kuka robotic arm that reproduced the angular movement (Figure 3). The Kuka KR5-SIXX-R650 system is used as the gold standard. Generally, optical motion analysis is more often used as the gold standard [13,14]. However, Kuka robotic arm is more accurate than the Optotrak optical motion capture system from Northern Digital, Waterloo, ON, Canada) [14] or the VICON, Oxford, UK, 612 [13] and it is a system that can perform various movements with fixed smartphones and Xsens at its extremity. We performed two different and complementary protocols (*cf.* Section 3.4.2 and Section 3.4.3) specifically designed to evaluate (1) the effect of the position of the smartphone on the Kuka robotic arm and repeatability of the measurement; and (2) the sensors and software accuracy performance (on smartphones and Xsens) compared to Kuka robotic arm, which is our gold standard, respectively.

#### 3.4.2. Protocol 1: Effect of the Position on Kuka Robotic Arm and Repeatability

In order to evaluate the effect of the device position on the Kuka robotic arm extremity and validate Protocol 2, the following Protocol 1 has been established. The Kuka robotic arm replicates measurements of angles from 0 to 180°, with a step of 5°, with stop of ten seconds at each position. This protocol was carried out 6 times for each axis (pitch and roll) and measurements were performed with only one filter, the OS filter of the iPhone 5S in iOS 7 operating system. It was executed under the following four conditions: (1) the hull of the smartphone is centered on the robot (iPhone 5S centered); (2) two smartphones, connected back to back with elastic, are centered on the robot arm (iPhone 5S with Galaxy Nexus); (3) smartphone is positioned on the arm at the location of its sensors (iPhone 5S centered on sensors); and (4) the smartphone is positioned in the opposite direction of the first condition (iPhone 5S in opposite direction).

#### 3.4.3. Protocol 2: Devices Performance Compared to Gold Standard

The aim is to compare, at the same time, accuracy performance from Smartphones and Xsens sensors, and to compare all previously described (Section 3.2) AHRS filters together. It has been done with respect to Kuka robotic arm, as gold standard, for the following static and dynamic conditions. For dynamic condition, the effect of velocity is also studied.

- The static state measure consists in replicating measurements of angles from 0 to 180°, with a step of 5° and a stop of ten seconds at each position. This protocol was carried out 6 times for each axis.
- The dynamic state measure consists in replicating measurements from 0 to 180°, with a step of 45° at rates of 20% and 50% of the maximum speed of the robot and with stop of ten seconds at each position. This protocol was also carried out 6 times for each axis and each speed.

### 3.5. Analysis

The accuracies of the smartphones and Xsens were assessed by computing, for each filter and devices in both protocols, the Root Mean Square (RMS) between angle estimation of devices and the gold standard. Gold standard was the theoretical set, whereas smartphone and Xsens measurements were variable set that we wanted to compare to this set. Thus, the RMS of the pairwise differences can serve as a measure of how far on average the error is from zero. RMS values were calculated for the six trials on three hundred samples. Note that such an analysis is widely used in the scientific literature [22,23,24].

A Kruskal–Wallis test was then used to evaluate the effect of position (in Protocol 1) by determining if RMS values from all the four conditions come from the same distribution and could be interpreted as similar. The Kruskal–Wallis non-parametric test is used when we are dealing with k independent samples (in our protocol, four samples of measures taken in four different positions) to determine if the samples come from the same distribution or at least one sample from a different distribution of others.

For performance comparison of sensors accuracy and its dependence on the filter, we have computed and compared, for each device and each filter, the RMS of the difference of the measurement and of the gold standard [22,23,24], for both static and dynamic protocols (Protocol 2). A Wilcoxon signed-rank test was used to evaluate the impact of velocity in the dynamic one. Then, a Kruskal–Wallis test was used to compare filters together for each device in each condition. We compared three samples of measurements taken with three different filters to determine if samples come from the same distribution. The best filter is then selected to evaluate Smartphone devices samples distribution relative to Xsens using, again, a Kruska–Wallis test.

## 4. Results

### 4.1. Protocol 1: Effect of the Position on Kuka Robotic Arm and Repeatability

To ensure the repeatability of the measure, a Kruskal–Wallis test was used for all six trials to compare the medians of RMS values from each condition to determine if there is a smartphone positioning effect on the robot arm. RMS was calculated for 27 angle values, avoiding values near 90° in order to prevent Gimbal lock effect. RMS values for roll and pitch, from manufacturer filter, are respectively presented in Table 1 and Table 2. For all conditions in the roll axis, no significant difference is observed (*p* > 0.01) in RMS for each trial against each other. For pitch axis, significant difference is observed (*p* < 0.01) in RMS for the condition where Smartphone is positioned on the arm at the location of its sensors.

**Table 1:** Effect of position, Root Mean Square (RMS) (variance | min–max) for roll in degrees.

	iPhone 5S Alone	iPhone 5S with Galaxy Nexus	iPhone 5S (Opposite Direction)	iPhone 5S (Centered on Sensors)
Trial 1	0.04 (0.004 | 0–0.23)	0.09 (0.011 | 0.02–0.32)	0.07 (0.009 | 0–0.34)	0.06 (0.010 | 0–0.43)
Trial 2	0.04 (0.004 | 0–0.21)	0.08 (0.012 | 0–0.32)	0.07 (0.009 | 0–0.33)	0.06 (0.010 | 0–0.41)
Trial 3	0.04 (0.004 | 0–0.26)	0.08 (0.009 | 0–0.28)	0.07 (0.008 | 0–0.35)	0.06 (0.010 | 0–0.38)
Trial 4	0.04 (0.004 | 0–0.26)	0.07 (0.009 | 0–0.28)	0.07 (0.007 | 0–0.30)	0.06 (0.010 | 0–0.40)
Trial 5	0.04 (0.005 | 0–0.27)	0.07 (0.009 | 0–0.26)	0.07 (0.008 | 0–0.31)	0.07 (0.010 | 0–0.42)
Trial 6	0.05 (0.006 | 0–0.27)	0.07 (0.008 | 0–0.26)	0.06 (0.008 | 0–0.37)	0.06 (0.011 | 0–0.44)

**Table 2:** Effect of position, RMS (min–max) for pitch in degrees. Bold typesetting indicates a statistically significant difference with the gold standard (*p* < 0.01).

	iPhone 5S alone	iPhone 5S with Galaxy Nexus	iPhone 5S (Opposite Direction)	iPhone 5S Centered on Sensors
Trial 1	0.05 (0.006 | 0–0.29)	0.09 (0.011 | 0–0.28)	0.05 (0.005 | 0–0.25)	0.12 (0.011 | 0–0.31)
Trial 2	0.05 (0.005 | 0–0.27)	0.08 (0.010 | 0–0.28)	0.05 (0.005 | 0–0.24)	0.11 (0.010 | 0–0.32)
Trial 3	0.05 (0.005 | 0–0.27)	0.08 (0.011 | 0–0.31)	0.05 (0.005 | 0–0.26)	0.11 (0.009 | 0–0.31)
Trial 4	0.05 (0.006 | 0–0.28)	0.08 (0.011 | 0–0.30)	0.04 (0.005 | 0–0.27)	0.11 (0.010 | 0–0.28)
Trial 5	0.05 (0.006 | 0–0.28)	0.08 (0.011 | 0–0.30)	0.05 (0.005 | 0–0.29)	0.11 (0.010 | 0.01–0.29)
Trial 6	0.05 (0.006 | 0–0.28)	0.08 (0.012 | 0–0.33)	0.04 (0.005 | 0–0.28)	0.11 (0.009 | 0–0.26)

### 4.2. Protocol 2: Devices Performance Compared to Gold Standard

#### 4.2.1. Static Protocol

**Figure 4:**
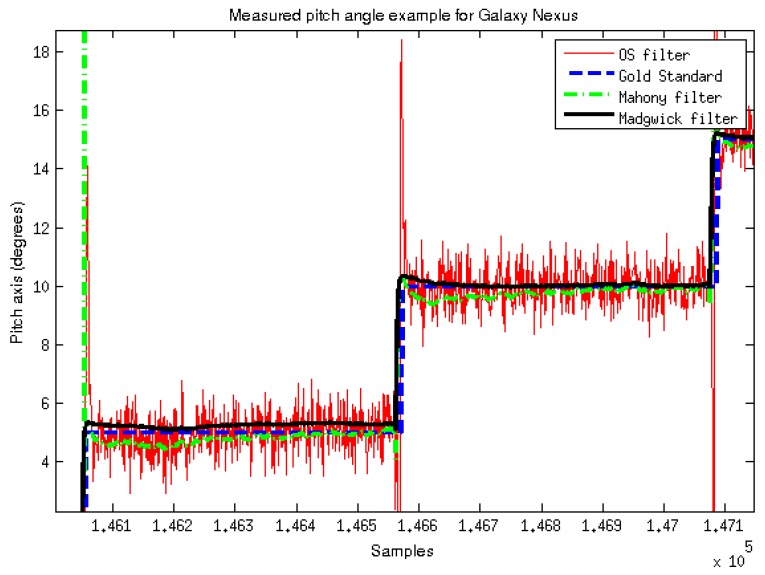
Signals measured during the static protocol for the second and the third targeted angles.

**Figure 5:**
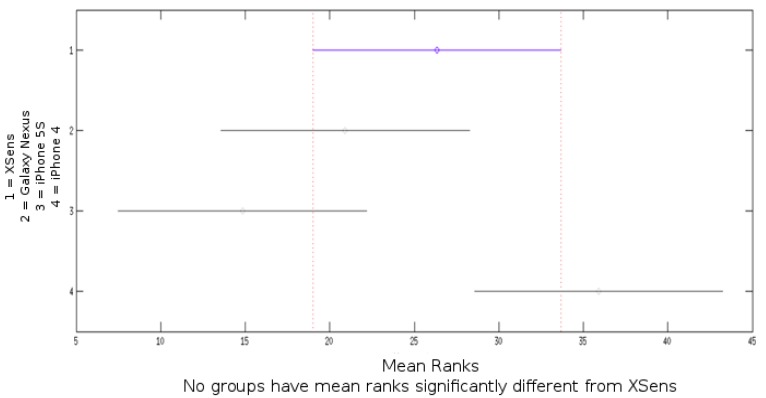
Kruskal–Wallis test result for Xsens roll and pitch Root Mean Square (RMS) compared to Galaxy Nexus, iPhone 5S and iPhone 4 obtained for the static protocol.

Figure 4 provides an example of a collected signal for each filter in the case of the Galaxy Nexus device. Table 3 shows the results for the four devices. As it can be seen in both the figure and table, for the Android Device, OS algorithm is noisier than the others (due to the kind of filter used by Android, more sensitive to integrative noise). RMS, between angle estimation of devices and the gold standard, and variance are presented. RMS and variance were calculated for 27 absolute angle values, avoiding values near 90° in order to prevent Gimbal lock effect in static protocol. Figure 5 shows the results from the Kruskal–Wallis test that compares Xsens RMS obtained for pitch and roll with smartphones devices.

Table 3 presents the values of the results obtained for both roll and pitch angles on the static protocol. We can note, from these results, that all the three smartphones give very good results. For roll angle, RMS is under 0.2° for all the filters and all the smartphones. There are no significant differences between the devices. iPhone 4 is better than the two others smartphones (it has been selected for this test as it includes the “old” generation of sensors of iPhone devices). Xsens sensors are, even with the manufacturer filter, over 0.2°. For the post-processing of Xsens data, the results are largely over the others (greater than 0.5°). We do not have any explanation, but we could infer that a different correction is perhaps done in the internal algorithm for the roll axis, as this effect is not present on the pitch one. As far as pitch angle is concerned, the results are almost the same. The results are also very positive, with a RMS value lower than 0.3° for all the sensors. iPhone 4 is still better than the others.

**Table 3:** Static protocol results, mean (min–max), for roll and pitch in degrees. Bold typesetting indicates a statistically significant difference between filters for a given manufacturer (*p* < 0.01).

		Roll		Pitch	
		RMS	Variance	RMS	Variance
**Nexus**	Manufacturer filter	0.16 (0.05–0.42)	0.36 (0.33–0.39)	0.21 (0.07–0.35)	0.42 (0.38–0.47)
	Madgwick	0.19 (0.07–0.42)	0 (0–0)	0.21 (0.06–0.62)	0.01 (0.01–0.01)
	Mahony	0.16 (0.05–0.38)	0 (0–0)	0.25 (0.05–0.50)	0.01 (0.01–0.01)
**iPhone 5S**	Manufacturer filter	0.15 (0.02–0.48)	0 (0–0)	0.13 (0.02–0.24)	0 (0–0.01)
	Madgwick	0.14 (0.03–0.47)	0.01 (0–0.01)	0.29 (0.05–0.55)	0 (0–0.02)
	Mahony	0.13 (0–0.50)	0.02 (0–0.09)	0.17 (0.03–0.29)	0 (0.01–0.01)
**iPhone 4**	Manufacturer filter	0.07 (0.01–0.18)	0.02 (0–0.17)	**0.08 (0.01–0.16)**	0.36 (0–1.12)
	Madgwick	0.10 (0.01–0.17)	0.55 (0–1.57)	**0.13 (0.02–0.63)**	0.08 (0–0.39)
	Mahony	0.12 (0.01–0.61)	0.09 (0–0.42)	0.09 (0.01–0.16)	0.56 (0–1.58)
**Xsens**	Manufacturer filter	0.22 (0.08–0.36)	0 (0–0.01)	0.22 (0.11–0.28)	0 (0–0.01)
	Madgwick	0.57 (0.02–3.44)	0.05 (0–0.62)	0.16 (0.05–0.29)	0 (0–0.03)
	Mahony	0.68 (0.02–5.45)	0.08 (0–1.26)	0.10 (0.03–0.18)	0 (0–0.02)

Concerning the influence of the filter, a Kruskal–Wallis test was done to compare, for a given manufacturer, the effect of the different algorithms. Only one significant difference was reported in the case of the iPhone 4 between the manufacturer filter and the Madgwick filter. There is no difference between the different filters for each device in the other cases.

Kruskal–Wallis test that compares Xsens manufacturer RMS results for pitch and roll with smartphones devices (Figure 5) indicate no significant differences between devices.

#### 4.2.2. Dynamic Protocol

**Figure 6:**
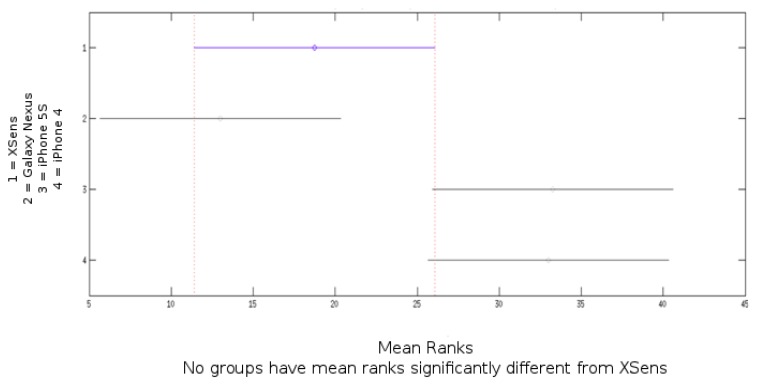
Kruskal–Wallis test result for Xsens roll and pitch RMS compared to Galaxy Nexus, iPhone 5S and iPhone 4 for the dynamic protocol at rate of 50% of the maximum velocity.

Table 4 and Table 5 show the results for the five devices for two different speeds during the dynamical protocol for, respectively, roll and pitch angles. Root mean square, between angle estimation of devices and the gold standard, and variance are presented. Figure 6 show result from the Kruskal–Wallis test that compare Xsens RMS result for pitch and roll with smartphones devices for rate of 50% of the maximum velocity.

**Table 4:** Dynamic protocol results, mean (min–max), for roll at rates of 20% and 50% of the maximum velocity in degrees.

		20%		50%	
		RMS	Variance	RMS	Variance
**Nexus**	Manufacturer filter	1.55 (0.64–3.51)	0.34 (0.31–0.38)	1.57 (0.81–3.43)	2.15 (0.59–4.37)
	Madgwick	3.36 (0.39–7.95)	0.02 (0–0.06)	2.84 (0.58–6.23)	0.90 (0.12–2.65)
	Mahony	3.56 (0.69–8.24)	0.03 (0–0.08)	3.44 (0.67–7.73)	0.99 (0.13–3.21)
**iPhone 5S**	Manufacturer filter	0.75 (0.32–1.33)	0 (0–0)	0.78 (0.32–1.29)	0 (0–0.02)
	Madgwick	8.05 (3.09–15.96)	0.73 (0.02–2.11)	8.70 (2.77–17.27)	0.99 (0.04–2.75)
	Mahony	2.42 (0.29–4.54)	3.60 (0–14.11)	4.16 (0.71–7.57)	6.09 (0.02–23.94)
**iPhone 4**	Manufacturer filter	3.57 (0.55–11.03)	0.55 (0.02–1.27)	3.52 (0.66–10.80)	0.48 (0.02–1.06)
	Madgwick	6.99 (0.91–17.92)	1.89 (0–7.56)	8.16 (0.95–20.35)	1.05 (0.01–3.66)
	Mahony	7.02 (0.95–17.97)	2.94 (0–11.74)	8.76 (1.02–21.35)	3.99 (0.01–15.90)
**Xsens**	Manufacturer filter	2.21 (0.84–4.29)	0 (0–0)	2.55 (0.84–4.99)	0.01 (0–0.03)
	Madgwick	10.39 (1.05–21.04)	1.28 (0–4.78)	10.51 (0.62–21.99)	2.24 (0–6.48)
	Mahony	3.93 (1.40–8.00)	7.49 (0–29.96)	6.94 (1.33–14.20)	26.79 (0–107.16)

**Table 5:** Dynamic protocol results, mean (min–max), for pitch at rates of 20% and 50% of the maximum velocity in degrees.

		20%		50%	
		RMS	Variance	RMS	Variance
**Nexus**	Manufacturer filter	2.29 (1.25–3.77)	3.74 (1.01–6.93)	2.00 (0.71–3.48)	1.42 (0.64–3.10)
	Madgwick	2.88 (1.61–4.78)	2.66 (0.79–5.41)	2.65 (0.77–4.15)	1.68 (0.03–3.53)
	Mahony	5.64 (0.66–15.25)	3.06 (2.03–5.19)	2.97 (1.07–5.17)	2.02 (0.09–3.02)
**iPhone 5S**	Manufacturer filter	1.94 (0.85–7.41)	0 (0–0)	1.94 (0.91–4.01)	0 (0–0)
	Madgwick	3.36 (0.34–7.41)	0.33 (0–0.91)	3.45 (0.25–7.26)	0.27 (0.04–0.76)
	Mahony	1.17 (0.14–3.18)	0.03 (0–0.06)	1.15 (0.48–2.69)	0.29 (0.01–0.71)
**iPhone 4**	Manufacturer filter	1.40 (0.37–2.20)	1.73 (0.07–2.33)	1.33 (0.32–2.18)	1.78 (0.07–2.45)
	Madgwick	1.97 (1.30–2.32)	0.02 (0–0.03)	1.93 (1.40–2.28)	0.11 (0.06–0.31)
	Mahony	1.12 (0.21–2.07)	0 (0–0.01)	1.05 (0.32–1.90)	0.07 (0–0.21)
**Xsens**	Manufacturer filter	0.87 (0.30–1.21)	0.02 (0–0.05)	0.94 (0.30–1.23)	0.04 (0–0.14)
	Madgwick	2.55 (1.02–4.10)	0.31 (0–1.20)	2.42 (0.65–4.19)	0.29 (0.01–0.95)
	Mahony	1.24 (0.19–2.94)	0.06 (0–0.11)	1.43 (0.25–3.22)	0.72 (0–2.24)

Table 4 and Table 5 show the results of the dynamic protocol at two different speeds. An important increase of the errors compared to the static protocol, whatever the speed, is observed. The algorithms have more difficulties exactly following the movements when the angular rate is too elevated during a very short duration (for instance 90° in less than one second). However, we can see that Xsens is better than the smartphones in that case, especially because the software includes a large acceleration mode that we chose and that change the range of measurement of the sensors (we cannot do such an operation on smartphones). Another point is that Xsens products are also certainly less sensitive to gimbal lock effect that will appear during such movements.

For this protocol, which aims to compare effect of the choice of the filter on the quality of the angle estimation, during dynamic protocol and for each device, there are no significant differences between filters reported by the Kruskal–Wallis test.

A Wilcoxon signed rank test was used to evaluate the impact of velocity. This test was performed on manufacturer filter, which is predominantly the best filters, considering that there are finally no significant differences between the filters. It can be observed that there are no significant differences between velocities for each device.

A Kruskal–Wallis test that compares Xsens manufacturer RMS result for pitch and roll with smartphones devices for rate of 50% of the maximum velocity (Figure 6) indicates no significant differences between devices. 

## 5. Discussion

In this paper, we propose an evaluation of different smartphone sensors and different manufacturer algorithm performances with the comparison to a gold standard, an industrial robotic arm and with a standardly used IMU in clinical measurement, the Xsens product. Effect of Smartphone position on the robotic arm during our protocol is first discussed. Then, accuracy performance compare to the gold standard is studied, as well as the effect of filter and effect of velocity and performance of smartphone in comparison with Xsens product.

### 5.1. Protocol 1: Effect of the Position on Kuka Robotic Arm and Repeatability

It can be observed that there is no significant difference between RMS error for roll axis, and only one significant difference for pitch axis in the case where the smartphone is fixed on the physical location of the sensors. Mean RMS for this condition is under 0.12°. To avoid this low bias, smartphone are always fixed on their center for all the studies. One can, however, consider that in the clinical context, our protocol can be considered as non-dependent on the position of the smartphone.

### 5.2. Protocol 2: Devices Performance Compared to Gold Standard and Xsens

#### 5.2.1. RMS and Variance

The four traces represent signal measurement, in degrees, for the Galaxy Nexus case in static protocol is shown in Figure 4. It illustrates the differences of variance of the signal between filters, and especially in the case of this Android device. It can be noticed that manufacturer filter provided by Android produce a bigger variance in the signal than other filters, but it produces the lower RMS mean results relative to other filters. Those RMS mean results are presented in Table 3 for the static protocol, where RMS values are calculated for 27 absolute angle values, avoiding values near 90° in order to prevent Gimbal lock effect and singularities in Euler angles representation. All RMS mean results are under 0.3° for both pitch and roll axis compared to the gold standard. Table 4 and Table 5 present RMS mean results for the dynamic protocol for two specific positioning velocities. The RMS mean values are higher than the results obtained in the static protocol, especially for Madgwick and Mahony filters. This might be due to Gimbal Lock effect that is not prevented in this protocol. It can particularly affect roll axis in the area of 90°, depending on yaw. An algorithmic solution can be used to solve these problems, but is not as effective as the use of quaternion. Manufacturer filters implement this type of algorithmic solution while other filters that we implemented did not. Xsens manufacturer filter RMS results are consistent with the accuracy provided by the manufacturer. 

#### 5.2.2. Context

These RMS and variance results have to be discussed in connection with the research context. For clinical context, results have to be under the acceptable error of 2.7° that is recently observed for passive range of motion with universal goniometer [25], which is the standard tool for this type of measurement. Passive range of motion is the movement of a joint through its range of motion without exertion by the subject, usually done by an examiner who moves the person’s body part manually. It could be compared with our static protocol. Moreover, in the same study [25], a smartphone application, which mimics goniometer with sensors, was also tested and the standard error measurement between testers is 1.4°. For acceptance of using such devices in clinical protocols, the error measurement between testers should be under five degrees [26] in upper extremity measurement and six degrees [26] or 5.5° [27] in lower extremity measurement for active range of motion. Active range of motion is the range of movement through which a patient can actively (without assistance) move a joint using the adjacent muscles, and could be compared with our dynamic protocol. In this present study, static measurement results are under acceptable clinical error for all filters, unlike the dynamic protocol results. Manufacturer filters are under acceptable errors, while others could be upper acceptable errors for dynamic protocol. However, this is, as explained above, due to the lack of compensation biases such as gimbal lock. Smartphones can therefore be considered sufficiently accurate tools for the clinical measurement of range of motion, but the filter effects have to be discussed.

#### 5.2.3. Impact of Velocity and Filter Effect

As we can see in Table 3 and Table 4, for static protocol, ranked in order of increasing precision filter is not homogeneous, in both pitch and roll, unlike dynamic protocol. However, the used of Kruskal–Wallis test for comparing filter results take into account the number of sample RMS result of each protocol. In this context, only one significant difference was found in the case of the iPhone 4 between the manufacturer filter and the Madgwick filter in case of static protocol, and there are no significant differences between filters reported for dynamic protocol. The effect of the filters, in this context, therefore is null. A Wilcoxon signed rank test was used to evaluate the impact of velocity during dynamic protocol on the quality of the measurement. Contrary to [23], no significant differences were found. However, our protocol is not exactly the same. RMS measurement is calculated during a static period after a wider angular movement than our static protocol. Finally, comparison between Xsens manufacturer filter and other manufacturer filters was done with a Kruskal–Wallis test. Xsens is a currently and widely used system for range of motion in clinical field [22,23]. No significant differences were found so it can be concluded that Smartphone range of motion results are at least comparable and similar to Xsens results. This confirms the previous conclusion: Smartphones can be used as a clinical tool to measure range of motion, and it can also be added that, for the three tested smartphones, there is no significant influence on the origin of the hardware sensors compared to Xsens.

## 6. Conclusions

In this paper, we have comprehensively evaluated the performance accuracy of smartphone sensors and algorithms. RMS mean values results for static protocol are under 0.3° for all Smartphones. RMS mean values results for dynamic protocol are more prone to bias induced by Euler angle representation. However, statistic results shows that in this context, there are no filter effect on results for both protocol and no hardware effect. In addition, the smartphone results can be compared to those of Xsens and especially to those of the gold standard, which is a Kuka robotic arm with a repeatability accuracy of ±0.02 mm. 

It can be concluded that built-in inertial sensors, in Smartphone, are reliable for clinical fields compared to standard tools like universal goniometer, in static protocol. In dynamic measurement, we were confronted with the limit of Euler angles representation for Madgwick and Mahony filters. To avoid this drawback, algorithms can be improved, such as it can be observed for manufacturer system filters. Moreover, in clinical fields, solutions could be made by following recommendations on the definitions of joint coordinate systems of various joints for the reporting of human joint motion [28]. Future research should take into account the medical field context.
